# Ethical considerations for the age of non-governmental space exploration

**DOI:** 10.1038/s41467-023-44357-x

**Published:** 2024-06-11

**Authors:** Allen Seylani, Aman Singh Galsinh, Alexia Tasoula, Anu R I, Andrea Camera, Jean Calleja-Agius, Joseph Borg, Chirag Goel, JangKeun Kim, Kevin B. Clark, Saswati Das, Shehbeel Arif, Michael Boerrigter, Caroline Coffey, Nathaniel Szewczyk, Christopher E. Mason, Maria Manoli, Fathi Karouia, Hansjörg Schwertz, Afshin Beheshti, Dana Tulodziecki

**Affiliations:** 1 School of Medicine, University of California, Riverside. 92521 Botanical Garden Dr, Riverside, CA 92507 USA; 2 School of Medicine, Medical Sciences and Nutrition, University of Aberdeen, Aberdeen, AB24 3FX UK; 3 Department of Life Science Engineering, FH Technikum, Vienna, Austria; 4 Heritage College of Osteopathic Medicine, Ohio University, Athens, OH USA; 5 Department of Cancer Biology and Therapeutics, MVR Cancer Centre and Research Institute, Calicut, India; 6 Department of Clinical Biochemistry, MVR Cancer Centre and Research Institute, Calicut, India; 7 Department of Molecular Medicine, Institute of Basic Medical Sciences, University of Oslo, Oslo, Norway; 8 Department of Anatomy, Faculty of Medicine and Surgery, University of Malta, MSD2080 Msida, Malta; 9 Department of Applied Biomedical Science, Faculty of Health Sciences, University of Malta, MSD2080 Msida, Malta; 10 Northwestern University Feinberg School of Medicine, Chicago, IL USA; 11 Department of Physiology & Biophysics, Weill Cornell Medicine, New York, NY USA; 12 Cures Within Reach, Chicago, IL 60602 USA; 13 Peace Innovation Institute, The Hague 2511, Netherlands & Stanford University, Palo Alto, CA 94305 USA; 14 Biometrics and Nanotechnology Councils, Institute for Electrical and Electronics Engineers, New York, NY 10016-5997 USA; 15 Department of Biochemistry, Atal Bihari Vajpayee Institute of Medical Sciences, New Delhi, India; 16 Center for Data-Driven Discovery in Biomedicine, Children’s Hospital of Philadelphia, Philadelphia, PA USA; 17 Deep Space Biology, San Francisco, CA USA; 18 School of Law, University of Aberdeen, Aberdeen, AB24 3UB UK; 19 Blue Marble Space Institute for Science, Exobiology Branch, NASA Ames Research Center, Moffett Field, CA USA; 20 Space Research Within Reach, San Francisco, CA USA; 21 Center for Space Medicine, Baylor College of Medicine, Houston, TX USA; 22 Molecular Medicine Program at the University of Utah, Salt Lake City, UT 84112 USA; 23 Division of Occupational Medicine at the University of Utah, Salt Lake City, UT 84112 USA; 24 Occupational Medicine at Billings Clinic Bozeman, Bozeman, MT 59715 USA; 25 Stanley Center for Psychiatric Research, Broad Institute of MIT and Harvard, Cambridge, MA USA; 26 Blue Marble Space Institute of Science, Space Biosciences Division, NASA Ames Research Center, Moffett Field, CA US; 27 Department of Philosophy, Purdue University, West Lafayette, IN USA

**Keywords:** Ethics, Medical research

## Abstract

Mounting ambitions and capabilities for public and private, non-government sector crewed space exploration bring with them an increasingly diverse set of space travelers, raising new and nontrivial ethical, legal, and medical policy and practice concerns which are still relatively underexplored. In this piece, we lay out several pressing issues related to ethical considerations for selecting space travelers and conducting human subject research on them, especially in the context of non-governmental and commercial/private space operations.

## Abstract (web summary)

New and dynamically changing opportunities for commercial/private and civilian spaceflight raise the need for an examination of how to ethically guide space industry and community. This Perspective explores such considerations with respect to space traveler selection and human subject research.

## Introduction

It has been over 50 years since the first human walked on the Moon. Since then, most commercialized spaceflights have been contracts granted to private companies by various governments to launch satellites, e.g., communication and GPS devices, into Earth’s orbit. In recent years, the definition of commercial spaceflight has expanded to include human transportation between Earth and habitats in Low Earth Orbit and future lunar or other extraterrestrial outposts. Once considered nearly impossible, commercial space travel is now a reality, due to rapid technological advancement in the private sector, large-scale investment from governments, and continued public interest. With the first crewed launch of SpaceX Dragon to the International Space Station (ISS), on May 30, 2020^1^, a new era of public-private spaceflight partnership has emerged. Private companies such as Boeing, Virgin Galactic, Axiom, Sierra Space, and Blue Origin now create a steep commercial demand for crewed spaceflight, both for technological and recreational purposes. NASA has further partnered with Axiom Space for commercial utilization of the ISS until the world’s first commercial space station is built by 2028^2^. There is currently a wide range of private/commercial spaceflights, ranging from suborbital flights lasting mere minutes to Axiom’s 1-2 week ISS missions to the first all-civilian orbital commercial spaceflight mission referred to as ‘Inspiration4’ (I4), comparable in duration (and health risks) to orbital shuttle missions^1–3^.

Historically, government-sponsored spaceflights were mission-based and geared towards national interests, e.g., technological leadership, improving national security, creating high-quality jobs, or advancements in research. After the Cold War era and the “Space Race,” government investment in space exploration declined. In contrast, the idea of commercial spaceflight continues to gain significant popularity and the economic opportunities linked to this are enormous. This has been accompanied by an influx of investment and the development of new technology, such as initial viable Reusable Launch Vehicle models by SpaceX^4^. Despite this rapid technology development, governmental regulation of commercial spaceflight lags behind. There are some requirements commercial spaceflight providers have to meet, including requirements concerning environmental safety, payload, payload re-entry, financial stability, and coverage for maximum probable loss^5^. Yet, there has been only limited regulation concerning the selection and training of non-government-sponsored astronauts and no formal oversight governing medical research on such crews, whether orbital or suborbital. The United Nations Office for Outer Space Affairs has collated national space laws relating to space activities from 42 countries^6^. While the regulations listed there cover a wide range of fields, such as objects in space, radiocommunication, and space activities, there is a general lack of health-associated regulations. This is a symptom of a bigger problem where there is little oversight of private sector spaceflight participants which, with Inspiration4, dearMoon^7^, and the upcoming Polaris program^8^, are already a reality.

An increase in commercial/private and civilian space missions with a more diverse crew may provide an opportunity to collect data on health issues in space. While these data could shape medical standards and improve treatment choices for prolonged spaceflight, their collection and management should require strict regulation. At the same time, as spacefarers shift from professionally trained astronauts to private individuals without rigorous preparation or with existing medical conditions, there is a need to refine selection criteria and training for non-governmental space travelers. Yet, despite their importance and urgency, these considerations have gone relatively unexplored^9^.

In this paper, we will lay out several pressing issues related to ethical considerations for selecting space travelers and research practices, especially in the context of non-governmental and private space operations. Although there are many other ethical issues related to space exploration – both commercial and otherwise^10–19^– our focus here is on ethical considerations regarding selection and human subject research. Note that while some of the issues we raise might be covered by guidelines, regulations, or law, this does not diminish the ethical considerations we discuss. A good example illustrating this point is NASA’s recent decision to unify the effective radiation exposure for male and female astronauts so as to not exceed 600 mSv, which translates to having to remain below 3% mean risk of cancer mortality above the non-exposed baseline mean, despite differences in male and female radiation-based cancer risk^20,21^. This decision is highly controversial, precisely because it is viewed by many as ethically problematic. Likewise, signing a consent form to undertake a certain activity (whether employer-mandated or not) does not make the proposed activity or consent process ethically unquestionable^21^. Indeed, our point here is that ethical considerations resulting from the increase in private and commercial spaceflight arise despite established rules and guidelines^22,23^.

A note on terminology: SpaceX, Axiom, and others refer to their travelers as ‘crew’. This has important consequences since the limited Federal Aviation Administration (FAA) guidelines^24^ that are applicable to non-professional astronauts are somewhat more stringent for crew than ‘ordinary’ spaceflight occupants. To bypass ambiguity, we prefer the term ‘spaceflight occupants’ (SOs), using it to refer to non-professional space travelers lacking substantial spaceflight training. Further, we use the terms ‘laws’ and ‘regulations’ to refer to binding law and the neutral terms ‘guidelines’, ‘recommendations’, and ‘policy’ to refer to non-binding guiding instruments.

## Considerations on governmental vs. non-governmental spaceflight

### Biological hazards of spaceflight

**Fig. 1:**
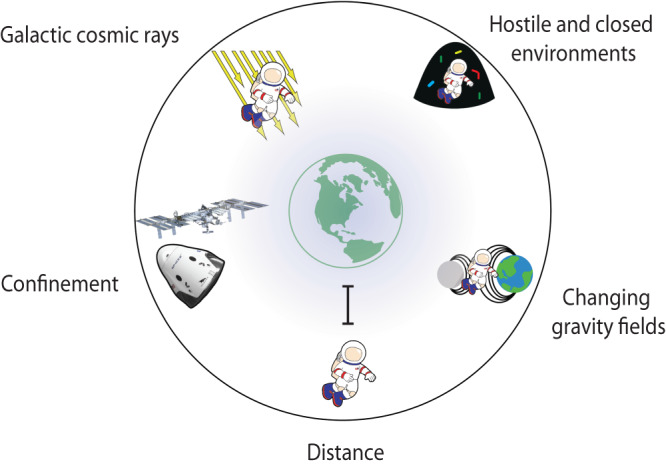
The Five Hazards in Space Contributing to Increased Health Risks. The figure exemplifies the main space flight hazards as used by NASA for the Human Research Road Map, such as distance, confinement, hostile and closed environments, galactic cosmic rays and space radiation, and changing gravity fields.

The selection of professional astronauts is highly regulated because space travel and habitation are demanding and dangerous. Current space missions to the ISS in Low Earth Orbit and future missions pushing the boundaries of human space exploration towards the Moon and Mars are characterized by exposure to space radiation, such as galactic cosmic rays (GCR), solar particle events (SPE), and trapped radiation^25^, changing gravity fields, acceleration/deceleration phenomena^26^, isolation, and confinement^27^ in a hostile and closed environment^28^ and, finally, the increasingly far distance from Earth (Fig. 1). Exposure to GCR/SPE is potentially the most significant single health hazard for Low Earth Orbit; for deep-space crewed missions beyond the Earth’s protective magnetic field this risk profile increases dramatically^28^. Recently, NASA increased an individual astronaut’s total career effective radiation dose (independent of age at exposure and sex) due to spaceflight radiation exposure to less than 600 mSv, translating into a mean risk increase of cancer mortality (REID) of below 3% above the non-exposed baseline mean^21^. This change was implemented following a study carried out under the auspices of the National Academies of Sciences^29^. However, these standards remain controversial, especially in light of predicted REID ranging between 6-10% for females exposed at ages 20, 40, and 60 years for a simulated Mars Mission^20^. Of note, most international partners use a higher career dose limit of 1,000mSV (independent of sex and age).

**Fig. 2:**
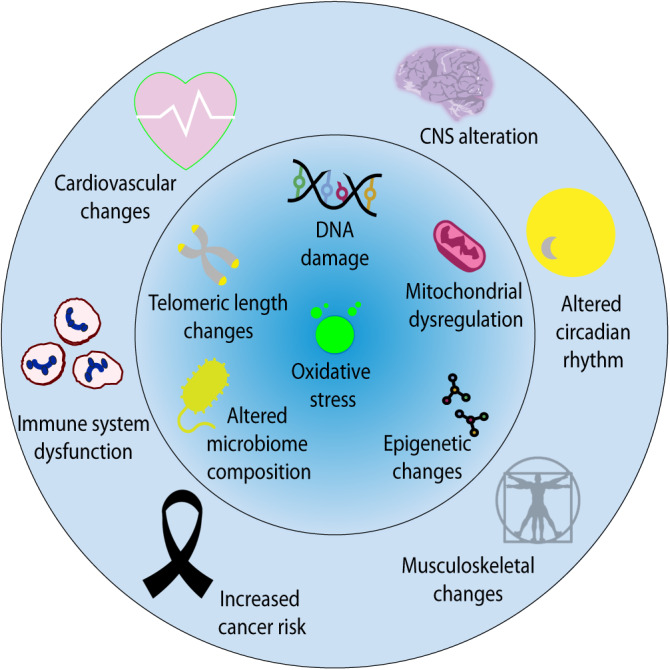
Biological and Health Features of Spaceflight. Space hazards, as outlined in Fig. 1, drive a diverse array of molecular and cellular changes observed during spaceflight, including DNA damage, oxidative stress, mitochondrial dysregulation, alteration in the microbiome composition, epigenetic changes, and telomere length changes. Such features will have the potential to induce pathophysiologic system changes affecting the central nervous system, the cardiovascular system, immune functions, musculoskeletal dynamics, the circadian rhythm, and cancer risk in SOs.

Health concerns and ethical considerations regarding commercial space travel are of great importance considering that the aforementioned potential hazards can severely interfere with many physiological processes, such as physiological homeostasis on a cellular and molecular level (Fig. 2), leading to microbiome shifts^30,31^, inducing dysregulated mitochondrial function^32^, and causing oxidative stress^33^. Furthermore, several space hazards cause DNA damage^28,30^, affect telomere length^30^, induce significant differences in DNA methylation, and alter accessibility of chromatin regions and specific DNA epitopes^30,34^. Such cellular and molecular effects lead to serious pathophysiology and health consequences^25^, including but not limited to: changes in the cardiovascular system including fluid shifts, orthostatic intolerance, reduced ventricle size, thrombus formation^28,35–39^, musculoskeletal defects defined as muscle atrophy and bone loss^30,40–42^, central nervous system alterations including fluid shifts, neurocognitive and psychiatric alteration and Space-Associated Neuro-ocular Syndrome (SANS)^43,44^, immune system dysfunction, initiation of malignant processes^45^ due to loss of DNA integrity, failed DNA repair mechanisms, mutations, and chromosomal rearrangements^46^. Nevertheless, while knowledge mounts, there remains a lack of data addressing individual differences among astronauts, such as age, sex, and genetic background, contributing to the challenges of accurately predicting radiation hazards and outcomes^28^.

### Ethical issues related to the dangers and selection of SOs

Given these significant spaceflight hazards, stringent selection criteria are important to protect astronaut and SO health and to help ensure that missions can be completed successfully. For instance, the ISS Multilateral Space Medicine Board provides guidance about travel to the ISS by private individuals^47,48^ and NASA has significantly strengthened its health-oriented recommendations and guidelines over the last two decades. Similarly, independent organizations such as the Aerospace Medical Association, have issued policies and guidelines taking into consideration not just the physical health of SOs but also their mental health^49^. There are not only expansive rules and guidelines at the selection and annual re-evaluation stage for astronauts, but also stringent health monitoring before, during, and after spaceflight^50^. However, with increasing commercial and private spaceflight opportunities, it is no longer just trained and pre-screened astronauts who travel to space. Since existing guidance in its various forms applies mostly to government-sponsored or -employed professional astronauts, the question arises as to who determines whether the potential health risks associated with spaceflight are acceptable or not in the case of SOs.

Current guidance on its own is not in a good position to resolve some of the issues that will arise in these new contexts. Both medical and scientific research communities as well as a number of governments have long and carefully deliberated about medical and ethical scenarios and questions that might arise during spaceflight. However, while some of their guidelines might apply to certain instances of, say, paying SOs (for example, private astronauts travelling to the ISS and therefore covered by ISS rules), there is still a relative dearth of discussion concerning how to extend these important deliberations to the new types of SO that we are likely to see in the coming decades.

Moreover, since existing law and policy frameworks were designed mostly with government-employed or -sponsored astronauts in mind, their scope does not always extend neatly to their non-governmental counterparts. For instance, federal space agencies form an employer-employee relationship with their astronauts. While this might be applicable to some kinds of potential commercial SOs, it will not be applicable to private SOs. For example, while the International Commission on Radiological Protection stresses the importance of radiological protection, in the U.S. the National Council on Radiation Protection and Measurements (NCRP)^51^ explicitly states that its purview is “NASA selection of astronauts for participation in space missions” and that “the measures suggested in this Report may be unique to NASA and not generalizable”. Although the refinements proposed by the NCRP to NASA’s shared decision-making framework do not directly pertain to commercial and private SOs, their relevance is evident and should be taken into consideration.

One might think that general documents of medical research ethics could provide some help here. For example, the World Medical Association’s (WMA) “Declaration of Helsinki” (DoH)^52^ is one of the most influential and important sets of ethical principles concerning medical research involving human subjects. While the WMA has no formal authority or binding status, it is still often expected that guidelines concerning research ethics will look to the DoH as a guiding document. However, the DoH is neither accepted by all countries nor uncontroversial. For example, Schüklenk and Ashcroft^53^ have highlighted both the “absence of a consensus over the actual content of the Declaration and its status”, and stressed its “continuing lack of a serious consultation with the relevant stakeholders”, while also emphasizing the more general “absence of a consensus among knowledgeable, well-intentioned bioethicists, scientists and political activists over the central issue of research ethics standards”. There is also a heated debate about whether there exists an international consensus opinion that violates and diverges from the DoH’s principles^54,55^. Macklin has argued that, even with revisions, the DoH “cannot resolve ongoing controversies”, since “it simply does not address other aspects of international research about which people disagree”^56^. Macklin has further stressed conflicts between the DoH and “official regulations promulgated by a federal agency, with enforcement mechanisms and sanctions for noncompliance” and pointed out that “it is hardly surprising that researchers, ethical review bodies, and governmental agencies do not consider the Declaration of Helsinki to be a necessary adjunct to the “official” Common Rule, which governs most federally funded research in the United States”^56^. Indeed, the US FDA first rejected the 2000 and later revisions, before eliminating all references to the DoH in 2006^57^. The situation is not dissimilar in many other countries and regions. Documents such as the DoH – regardless of its controversies – are much too general to resolve particular ethical conundrums related to human space travel. Thus, while they might provide some necessary restrictions and sometimes even positive guidance, the question of how such general principles apply to specific and concrete cases, especially when there is little precedent, requires further examination. This of course is just one reason why agencies around the world spend enormous efforts on crafting guidelines and recommendations about the ethics of human subject research in space. But who will do the same for those space travelers to whom these do not apply?

The relevant international legal framework, i.e. the five UN Space Treaties, do not specifically address the health of astronauts or other spaceflight participants^6^. The Outer Space Treaty (OST) of 1967 introduces the concept of “envoys of mankind” for astronauts^58^; the Rescue and Return Agreement (RRA) extends this protection to “personnel of spacecraft”. However, neither of these terms is clearly defined and terminological inconsistencies have led to a broad interpretation, suggesting that the Agreement extends to human life in outer space or spaceflight generally, covering both professional and non-professional space travelers, including tourists. However, these agreements were drafted at a time when activities such as space tourism were unforeseen, posing challenges in predicting and regulating health concerns for modern space activities. Thus, these agreements lack explicit health regulations for individuals that are part of the private space industry and, even if such regulations existed, enforcing them would be challenging due to the absence of robust enforcement mechanisms in international law.

**Fig. 3:**
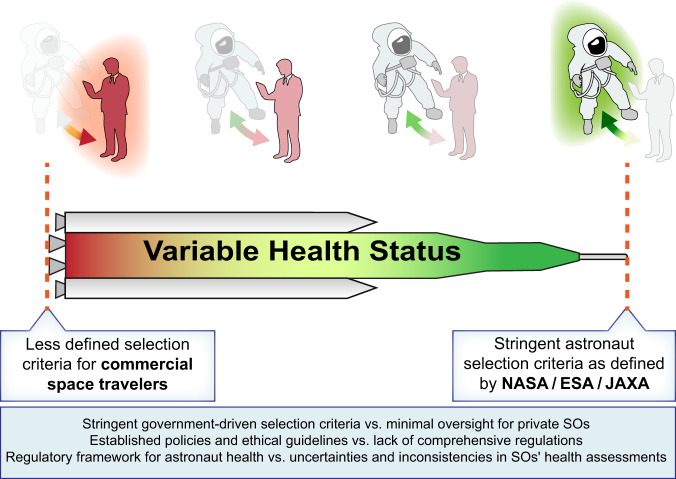
Differential Approaches to Selecting SOs. Stringent selection criteria (green) for government-sponsored astronauts (i.e., NASA, ESA, JAXA), including a hard stop mechanism are depicted on the right of the schematic (right dashed red line). Less defined commercial space traveler criteria for civilians (red) finally leading to rejection (left dashed red line) are indicated. The green-red color-coded middle area symbolizes the health-related risks, where the array of potential commercial space selection criteria is located.

To address issues such as these at the US-level, the FAA^59^ and NASA entered a Memorandum of Understanding in June 2012, to coordinate standards for commercial government or non-government astronaut transport to and from Low Earth Orbit and the ISS. The goal was to foster both public and crew safety, avoid conflicting rules and guidelines, as well as to provide a framework for the American space industry. Despite this, the clearance of ‘ordinary’ SOs for spaceflight is currently the responsibility of commercial providers, with limited oversight, and a lack of standardized screening procedures and protocols^60^. The US is currently the main country providing such guidance in the form of the FAA *Recommended Practices for Human Space Flight Occupant Safety*. However, due to the limits of FAA jurisdiction, these guidelines only apply to launch and reentry^61^. Further, the FAA guidance comes not in the form of mandatory requirements but rather as (minimal) recommendations, suggesting merely that “[p]roximate to flight, the operator should require each space flight participant to consult with a physician, trained or experienced in aerospace medicine, to ascertain their personal medical risks from the space flight profile and vehicle” (2023: B. 4.4.2). It also explicitly states that “[t]his document does not include any specific medical criteria that would limit who should fly in space as a space flight participant.” (2023: A. 6.1). Thus, given that medical consultation is only a recommendation, in principle anyone capable of giving written informed consent can fly, regardless of their health profile, as long as they meet specified spaceflight operator criteria (Fig. 3). This raises a number of issues.

First, if an SO fails to be cleared by one physician, can they get clearance from a more lenient provider elsewhere?^62^ As Langston points out, “[m]edical forum shopping is a foreseeable ethical and legal concern where regulatory standardization is lacking or is inconsistent between jurisdictions… [which] could lead to increased risk of harm for the individual (in-flight/post-flight), spaceflight and crew, as well as uninvolved third parties”^63^. This scenario parallels a situation seen in the Federal Motor Carrier Safety Administration^64^, where a disqualified driver can apply for a resolution if a disagreement exists in regard to the medical qualification exam. However, to oversee potential medical forum shopping, all results from medical examinations have to be entered into the centralized National Registry, preventing the continued medical examination until a desired result is achieved and certified.

Second, there might be tension between the potential clearance requirements desirable for a commercial spaceflight operator or provider – who might have an interest in lenient requirements to sell as many seats as possible to the limited pool of individuals who can afford them – and those desirable from the health standpoint of a particular SO. One might argue that SOs should undergo relevant medical consultations and decide on the risks they are willing to take, similar to making choices about risky adventures on Earth. So why should they not be in a position to make similar decisions for space? Nonetheless, while legal and social norms allow people to engage in some risks, the same norms do not give blanket allowance for people to do anything they want, instead exercising various degrees of paternalistic oversight both in people’s private and public lives (for an overview of paternalism and its issues, see Dworkin 2020)^65^. For example, many governments require drivers and passengers to wear seatbelts, they require motorcyclists to wear helmets, forbid the sale of certain drugs, they forbid people to enter into certain kinds of contracts (for selling organs, gambling debts, etc.). Questions thus arise about the appropriate degree of paternalistic oversight for space travel, as well as questions about who has the power to enforce relevant policies and regulations. A further complication – and again one that speaks in favor of increased guidance or regulation – is that compromised SO health might also affect fellow passengers. In fact, the September 2023 revisions of the FAA recommendations now recognize this problem at least partially, stating that “[m]edical consultation for space flight participants is recommended … to help prevent them from endangering other occupants …. [and] commercial operators will be challenged to control hazards to space flight participants from other space flight participants with medical conditions.”^61^ This is, of course, also a problem with respect to various forms of ground and air transportation; however, it is exacerbated in the case of space travel. For one, providing assistance in space is more difficult than on the ground or in the air, thus putting fellow travelers in a position in which they are more likely to compromise their own safety during the course of providing help, especially in the absence of mandatory training. Further, space travel has inherent limits about the amount and kind of medical equipment that can be transported and effectively used in a spaceflight environment.

Third, even if stringent screening requirements are in place, substantial uncertainties remain regarding the various health consequences of space travel, making it virtually impossible for specific individuals to understand what their actual health risks are, even if advised by someone “trained or experienced in aerospace medicine”^61^. Current data insufficiently address individual differences among astronauts such as age, sex, and genetic background^66^, which translates into significant uncertainty in predicting individualized space radiation hazards and outcomes for both astronauts and SOs^67^. In addition, while it is known that individuals metabolize drugs and supplements differently in space^68–70^, little to nothing is known about the physiology underlying these changes. Limited available data^70^ suggests that even relatively common prescription drugs might work differently in space. Furthermore, the reduced and often unknown stability of pharmacologic ingredients and supplements over the course of exploration-rated space missions becomes even more critical^70^. Specially prepared space medications, appropriate repackaging to improve pharmaceutical stability, and more insight into individual pharmacokinetics are needed to supply both astronauts and SOs with effective pharmaceuticals and/or other treatment options.

All of these considerations strongly suggest that some guidance and/or oversight with respect to potential SO screening and clearance would be highly beneficial (Fig. 3). In fact, this is not just so for the SOs, but also for the commercial providers who agree to transport them. While SOs are currently required to sign liability waivers, the legal status of these waivers is unclear, and so binding rules might also provide a way for providers to indemnify themselves against future lawsuits^71^. However, such rules should be sensitive to the diversity and variety of potential spaceflight endeavors: there is a difference between a private citizen enjoying a suborbital flight and a commercial crew member spending prolonged time in space for research purposes, perhaps on a commercial space station.

### Human subject research

As our technical and research abilities evolve, so do the ethical considerations for using human subjects for research. While NASA estimated the chance of survival of the first mission to the Moon (Apollo 8) at 50-50, such odds for harm would not be accepted for any present-day study involving human subjects (recollection quoted from Ref. ^72^). NASA’s ethical principles are defined to ensure human research subject welfare and minimize health risks. Further, research protocols can be implemented only if a risk/benefit analysis demonstrates that the risks to the subjects are reasonable in relation to the anticipated benefits and the expected importance of new knowledge. This will become even more important for missions to Mars^73^. In addition to the prevention of direct harm, other important considerations include protecting privacy, ensuring strict data security, and maximizing the positive social impacts of research.

Human Subject Research projects in space, supported or otherwise subject to regulation by any US federal department or agency, are strictly regulated under the Code of Federal Regulations (14 CFR pt 123069). Based on this, the implementation, procedures, and requirements to conduct space-related research involving human subjects is tightly controlled within a binding framework. NASA Institutional Review Board (IRB) committees review such research proposals to guarantee enforcement of these policies and the ethical, safe, and equitable treatment of human research subjects. In addition, the Office of Research Assurance ensures that all activities comply with applicable federal regulations and guidelines, ensuring that human subject welfare and minimal health risk are prioritized for all decisions. Such analysis will not take into account potential long-term effects on public policies. An interesting variation from non-NASA research protocols is the requirement that the responsible flight surgeon maintains the duty to intervene and terminate ongoing research if the health and welfare of astronaut research subjects is in question.

While the NASA IRB process adheres to standard practices for IRBs, it has unique aspects focused on human subjects’ well-being beyond typical standards. The highest level of concern involves human subject research studies involving genetic testing. NASA defines genetic testing based on the Genetic Information Nondiscrimination Act^74^. Studies involving genetic testing in human subjects are deemed of the highest concern and automatically categorized as “greater than minimal risk”, according to NASA^75^. These studies require additional measures to protect the research subjects, including policies that prohibit the public release of genetic data without prior approval from the individual or their direct family members, in accordance with NASA policy. NASA enforces strict rules as genetic data must be stored separately, and cross-referencing is forbidden without IRB approval. After genetic testing, all electronic data is deleted and given solely to NASA. However, commercial institutions or providers using services at NASA facilities to launch a space vehicle do not have to adhere to such rules in the same way.

While the above-described scenarios are focused on NASA, the processes for international ISS partner research are not demonstrating significant national differences. In general, plans for research involving human research subjects are carefully examined by the ethics committee of the researcher’s university or institute, the space agency proposing the research, and the space agency of the astronaut subject. Furthermore, the Human Research Multilateral Review Board (HRMRB), comprising the representatives of space agencies involved in the ISS (NASA, ESA, CSA, JAXA), is tasked with investigating and reviewing ethical matters on the ISS. For all research conducted on an astronaut, the HRMRB looks at whether the safety and health of the astronaut are assured and whether the appropriate ethical considerations have been made. As necessary, the HRMRB then issues recommendations or modification requests. While government-sponsored human subject research is strictly regulated, it is still unclear whether future commercial SOs will be covered under such regulations, or if adherence to government regulations needs to be amended to reflect the new reality of space human subject research.

### Ethical considerations

To date, our knowledge regarding the effects of spaceflight on humans, as well as the efficacy and safety of select medical interventions and pharmaceuticals in space, comes from government-sponsored missions, a small number of astronauts, and, more recently, civilians (Inspiration4, MS-20, Axiom-1)^76,77^. Increasing this type of knowledge is vital to ensuring the safety of future astronauts and SOs, especially considering the limited opportunities for medical treatment in space. It is therefore likely that astronauts and SOs will need to monitor, diagnose, and treat themselves at least part of the time. Increased commercial and private spaceflight opportunities will bring with them an expanding diversity of SOs with different health profiles, most of them likely not in the same physical and psychological shape as highly trained career astronauts^78–80^. By necessity, increasing knowledge about the effects of the spaceflight environment on human molecular biology, physiology, and psychology, as well as on the chemical composition of pharmaceuticals, involves human subject research. With the success of the recent Inspiration4 mission in defining a wide range of biomedical data, the role of commercial and private SOs in such research is likely to increase.

**Fig. 4:**
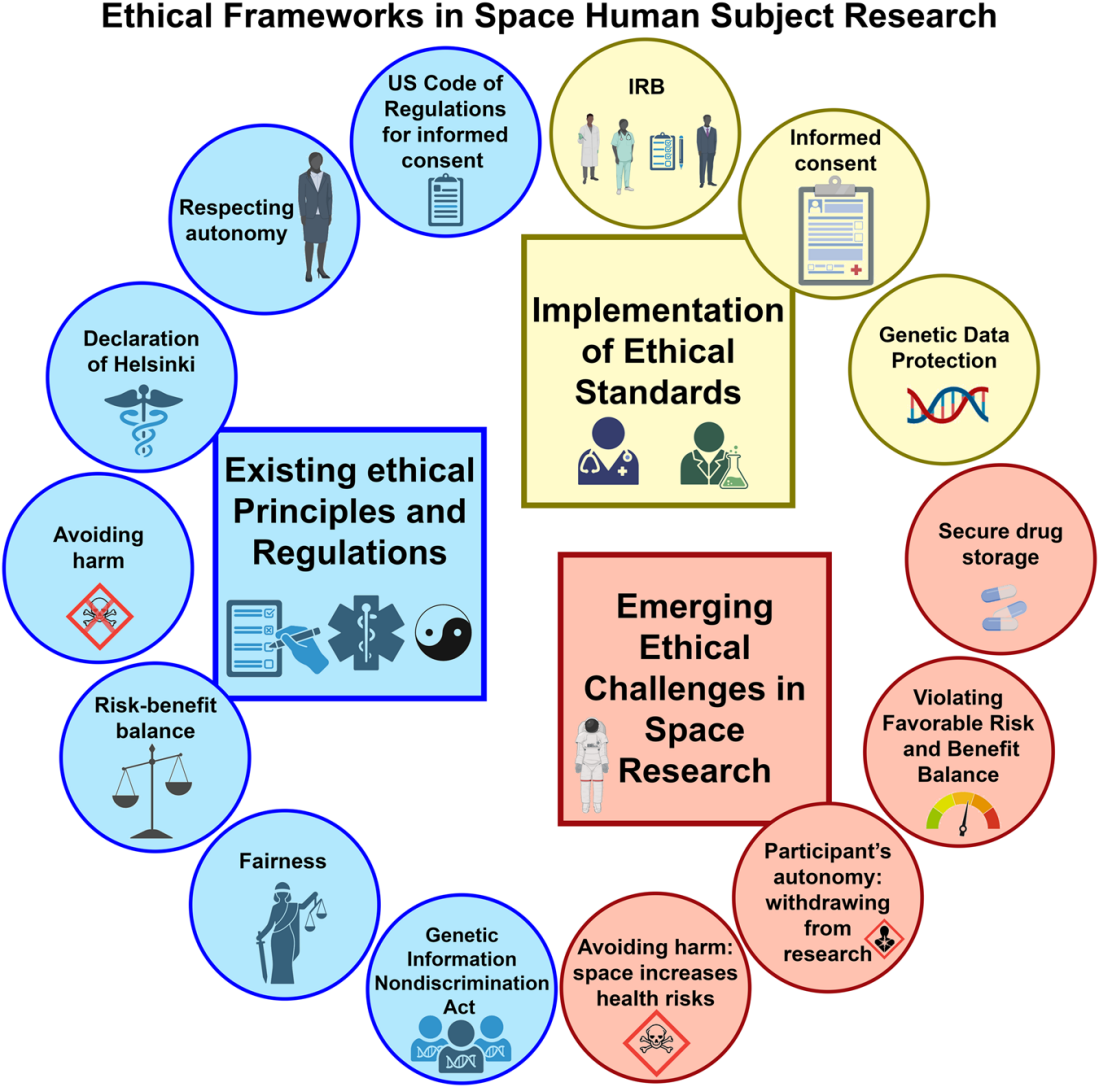
Human Subject Research Ethical and Operational Guidelines. A schematic for the ethical framework for space human subject research. This framework defines the key existing principles and regulations that currently exist in human research (in blue), the implementation of these ethical standards in the clinic and research laboratory (in yellow), and the challenges that should be considered and will arise for human research in space (in orange).

As previously outlined, government-sponsored spaceflights adhere to stringent criteria with respect to human subject research, through their respective IRBs, ethics committees, and groups. In the case of Inspiration4, research was conducted through Weill Cornell Medicine, the Translational Research Institute for Space Health of the Baylor College of Medicine^81^, California Institute of Technology, and Massachusetts Institute of Technology and underwent similarly rigorous approval processes^82^. As the general standard for human subject research requires compliance with ethics approvals, it is expected that commercial spaceflight-based human subject research will continue to follow these and other applicable internationally ratified processes (Fig. 4).

However, commercial spaceflight also raises new ethical issues. In the past, human subject research in space was mostly a secondary concern of overall missions whereas now missions are increasingly conducted explicitly for this purpose. Ethical recruitment for biomedical research requires informed consent. For space travel, this requires participants to have a good understanding of the space environment, the nature of associated environmental hazards, as well as the potential side effects of any drugs under study^51^. But to what extent is such consent really possible with respect to space travel?^83^ Exposure to high radiation, microgravity, and galactic rays may compromise participant safety in a number of unknown ways. Further, the effect of radiation and other extra-terrestrial factors on the chemical composition of pharmaceutical ingredients is largely unknown, thus making it especially difficult to inform potential subjects about likely side effects. The US Code of Regulations for informed consent requires a “description of any reasonably foreseeable risks or discomforts to the subject” (Title 45, A.A. Part 46.116, b2^82^). In the case of space travel, where many or most risks are unknown, it is unclear to what extent – if at all – this requirement can be met. Thus, there is a need to incorporate new and emerging ethical issues into existing frameworks in ways that are based on the same underlying ethical principles that gave rise to these and similar regulations in the first place and that are applicable to space travel. This is all the more urgent since civilians are already involved in such research^51^. Note also that the aforementioned problems are exacerbated for non-astronaut SOs: most of the existing data is not from a random population, but rather from an elite force markedly dissimilar to average citizens in terms of training, age, health, and biomedical and behavioral profiles. Additionally, it has been established that even highly trained individuals respond quite differently to the zero-gravity environment^84^. Moreover, existing research was conducted on a population with a high degree of sex, gender, and ethnic homogeneity and relatively low diversity. Currently, it is therefore unclear to what extent this research generalizes to SOs beyond the relatively narrow population that has been studied so far.

Furthermore, principles of health ethics encompass avoiding harm, beneficence, achieving a favorable risk-benefit balance, respecting autonomy, fairness, and fidelity^85^. Terrestrial clinical trials rely on large participant numbers, secure drug storage, and timely sample collection, which are difficult to replicate in space. How to ensure compliance with these principles in space thus remains uncertain. There are also some concerns about individual principles: For example, human subject research in space may violate a participant’s autonomy, should the individual decide to withdraw from the study. In terrestrial medical research, subjects may at any time withdraw their consent, even if the study is already in progress. Such withdrawal, however, is much more complicated in space. Even if individuals are able to drop out, return to Earth will not be immediate. There is also no way for individuals to withdraw from the possible (unknown) long-term effects of the spaceflight environment, and any consent thus involves not just consent to the study itself, but consent to any future effects that might result from spaceflight activities. This might also lead to violating the Avoiding Harm principle, since remaining in space is unhealthy for participants, especially if the desired withdrawal occurs as the result of unforeseen health problems. In turn, these conditions might then violate the Favorable Risk and Benefit Balance, due to the changing circumstances during the course of research. In NASA’s case, the responsible flight surgeon can interrupt trials. However, in the absence of official, government-sanctioned flight surgeons with extensive spaceflight experience, who assumes the equivalent responsibilities for SO healthcare, ensuring subjects’ best interests are met?

These problems represent only a small subset of issues, demonstrating the urgent need for guidance on non-government space research on human subjects (for further issues relating to privacy and behavioral health, see^86,87^). Such research should follow strict ethics committee review and otherwise meet the same stringent standards that NASA, ESA, and JAXA currently adhere to^88^, including following suggestions by international medical research bodies, such as the DoH, The Council for International Organizations of Medical Sciences’ International Ethical Guidelines for Health-Related Research Involving Humans^89^, and others.

## Conclusion

We are entering an exciting new era of space exploration previously only thought of as science fiction. Commercial/private and civilian spaceflights, such as Inspiration4 and the Polaris missions are no longer just a possibility but a reality. Exciting as these opportunities are, they also come with the burden of ensuring that future space travel will be as safe and ethical as possible. While governmental oversight has historically governed space activities, the emergence of non-governmental initiatives calls for unified ethical guidelines, safeguarding human well-being during selection, research, and decision-making in space. Since non-governmental outfits are not bound by the same rules in the same way, we as a community must ensure that the guidelines we set will guide space exploration according to the highest ethical and medical standards for humans that are currently possible, including selection of SOs, medical research ranging from human subject research to discussion of in-flight triage decisions. The added difficulties of non-universal terminology referring to different kinds of space travelers and of making guidelines sensitive to the diversity and variety of potential spaceflight endeavors will add further layers of complexity. The earlier and better any such guidelines can be implemented, the better the chances that space travel can be performed according to the safest and most optimal standards.
